# Bioinformatics analysis and identification of genes and molecular pathways in steroid-induced osteonecrosis of the femoral head

**DOI:** 10.1186/s13018-021-02464-9

**Published:** 2021-05-20

**Authors:** Tianye Lin, Weijian Chen, Peng Yang, Ziqi Li, Qiushi Wei, Du Liang, Haibin Wang, Wei He, Qingwen Zhang

**Affiliations:** 1 The First Clinical Medical College, Guangzhou University of Chinese Medicine, Guangzhou, 510405 Guangdong China; 2 Guangzhou University of Chinese Medicine, Guangzhou, 510405 Guangdong China; 3 Guangzhou Orthopedic Hospital, Guangzhou University of Chinese Medicine, Guangzhou, 510045 Guangdong China; 4 The Lab of Orthopaedics of Chinese Medicine of Lingnan Medical Research Center, Guangzhou University of Chinese Medicine, Guangzhou, 510405 Guangdong China; 5 Department of Joint Orthopaedic, the Third Affiliated Hospital, Guangzhou University of Chinese Medicine, Guangzhou, 510405 Guangdong China; 6 Institute of Orthopedics, Guangzhou University of Chinese Medicine, Guangzhou, 510405 Guangdong China

**Keywords:** Osteonecrosis of the femoral head, Differentially expressed gene, Enrichment analysis, Peripheral blood, Cartilage

## Abstract

**Background:**

Steroid-induced osteonecrosis of the femoral head (ONFH) is a common hip joint disease and is difficult to be diagnosed early. At present, the pathogenesis of steroid-induced ONFH remains unclear, and recognized and effective diagnostic biomarkers are deficient. The present study aimed to identify potentially important genes and signaling pathways involved in steroid-induced ONFH and investigate their molecular mechanisms.

**Methods:**

Microarray data sets GSE123568 (peripheral blood) and GSE74089 (cartilage) were obtained from the Gene Expression Omnibus database, including 34 ONFH samples and 14 control samples. Morpheus software and Venn diagram were used to identify DEGs and co-expressed DEGs, respectively. Besides, we conducted Kyoto Encyclopedia of Genome (KEGG) and gene ontology (GO) pathway enrichment analysis. We construct a protein-protein interaction (PPI) network through GEO2R and used cytoHubba to divide the PPI network into multiple sub-networks. Additionally, quantitative real-time polymerase chain reaction (qRT-PCR) was performed to verify the bioinformatics analysis results.

**Results:**

A total of 118 intersecting DEGs were obtained between the peripheral blood and cartilage samples, including 40 upregulated genes and 78 downregulated genes. Then, GO and KEGG pathway enrichment analysis revealed that upregulated DEGs focused on the signaling pathways related to staphylococcus aureus infection, leishmaniasis, antigen processing, and presentation, as well as asthma and graft-versus-host disease. Downregulated genes were concentrated in the FoxO signaling pathway, AMPK signaling pathway, signaling pathway regulating stem cell pluripotency, and mTOR signaling pathway. Some hub genes with high interactions such as CXCR1, FPR1, MAPK1, FOXO3, FPR2, CXCR2, and TYROBP were identified in the PPI network. The results of qRT-PCR demonstrated that CXCR1, FPR1, and TYROBP were upregulated while MAPK1 was downregulated in peripheral blood of steroid-induced ONFH patients. This was consistent with the bioinformatics analysis.

**Conclusions:**

The present study would provide novel insight into the genes and associated pathways involved in steroid-induced ONFH. CXCR1, FPR1, TYROBP, and MAPK1 may be used as potential drug targets and biomarkers for the diagnosis and prognosis of steroid-induced ONFH.

## Introduction

Osteonecrosis of the femoral head (ONFH) is a common disease of the hip with a higher incidence in elderly patients, and the most common clinical symptom is severe pain [1]. There are about 150,000–200,000 new cases in China each year [2], and about 20,000 new cases in the USA each year [3]. The pathological features of steroid-induced ONFH include decreased blood supply to the hip joint, the collapse of the femoral head, and accumulation of microfractures without continuous remodeling [4, 5]. Besides, ONFH can cause rapid destruction and dysfunction of the hip joint, and approximately 65–70% of patients with advanced ONFH require total hip replacement [6]. The pathogenesis and molecular mechanism of steroid-induced ONFH remain unclear, and there are few effective prevention and early treatment methods.

Studies have indicated that successful results can be obtained when joint-preserving surgery is used in the pre-collapse stage of steroid-induced ONFH [7]. After the femoral head necrosis collapses, cartilage wrinkles and the hip joint space gradually narrows. Meanwhile, the failure rate of joint-preserving treatment is high. Therefore, searching for new biomarkers in the blood is of great clinical significance for the diagnosis of steroid-induced ONFH, as well as the further early diagnosis and treatment of ONFH. Some genetic factors are considered key factors in the progress of steroid-induced ONFH, such as peroxisome proliferator-activated receptor γ, matrix metallopeptidase 9, and SMAD family member 3 and gremlin 1 [8–10]. Currently, microarray analysis using a high-throughput platform is promising and efficient for exploring the molecular mechanisms of diseases and identifying useful biomarkers for diagnosis and prognosis of diseases [11, 12]. However, there is little comprehensive research on steroid-induced ONFH with high-throughput platforms. During the development of steroid-induced ONFH, the cartilage on the surface of the femoral head and the labrum of the acetabulum are significantly damaged. Degeneration of cartilage on the surface of the femoral head and tearing of the labrum enhance the instability of the hip joint and accelerate the development of steroid-induced ONFH [13]. Prevention and early treatment of hip cartilage damage may slow the development of steroid-induced ONFH and relieve hip dysfunction. However, there are few studies on the molecular mechanism of steroid-induced ONFH hip cartilage damage. Therefore, it is hypothesized in this paper that differentially expressed genes co-exist in the peripheral serum and cartilage of ONFH patients; this can be used for early diagnosis and observation of disease progression.

In this study, hub genes are screened out from the differences expressed genes (degrees) using bioinformatics methods based on the National Center for Biotechnology Information (NCBI http://www.ncbi.nlm.nih.gov/geography/) from the Gene Expression Dataset GSE123568 (peripheral serum) and GSE74089 (hip articular cartilage) Comprehensive (GEO) database. Quantitative real-time PCR (qRT-PCR) was conducted to validate the gene expression profiling results based on an independent sera sample of eight patients with ONFH and twelve healthy controls. The present study aimed to identify potentially important genes and signaling pathways involved in ONFH and investigate their molecular mechanisms.

## Materials and methods

### Microarray data source

The gene chip data related to femoral head necrosis (GSE123568, GSE74089) is downloaded in the GEO (gene expression omnibus) database (https://www.ncbi.nlm.nih.gov/geo/). GSE123568 chip data contains 10 normal patient serum samples and 30 steroid-induced femoral head necrosis serum samples. Besides, the GSE74089 chip data is composed of hip cartilage specimens from 4 NFH patients and 4 healthy controls. All the above data sets are based on GPL15207 and GPL13497 platform GEO database downloaded, and the information of the above two data sets are detailed in Table 1.

**Table 1:** Two datasets used for gene expression profiles analysis in NFH

GEO ID	Author	Platform	Samples	Type	Year	Omics
**GSE123568**	Zhang Y	GPL15207	NFH to normal = 30:10	Peripheral serum	2019	mRNA
**GSE74089**	Ruiyu L	GPL13497	NFH to normal = 4:4	Hip articular cartilage	2016	mRNA

### Differentially expressed genes analysis

GEO 2R (http://www.ncbi.nlm.nih.gov/geo/geo2r/) is an online analysis program based on the R language provided by the GEO database (R 3.2.3 version) [14]. The Bioconductor R package in GEO2R is used to perform background correction, normalization, and expression value calculation on the GSE123568 and GSE74089 data; the limma 3.26.8 package is adopted to calculate the differential genes between the two groups and derive the GEO2R processed data. Set *P*< 0.01, adjust *P* value < 0.01, and the criteria for screening differential genes is that the expression change range is greater than or equal to twice (|log2 FC|≥1.0). Besides, log2FC≥1.0 indicates upregulation of gene expression; log2FC ≤− 1.0 suggests downregulation of gene expression. Finally, the differentially expressed genes of the femoral head necrosis group and the healthy control group, namely the differentially expressed genes (DEGs) of femoral head necrosis, were obtained. Using the online analysis website ClustVis (https://biit.cs.ut.ee/clustvis/) [15], the heat map and cluster analysis of the selected differential genes are completed, and the -log10 conversion is performed for the adjust *P* value in the data processed by GEO2R. Then, according to log2 FC, -log10 (adjust *P* value) was divided into groups of upregulated genome, downregulated genome, and non-statistically different genome. Next, the processed data is imported into GraphPad Prism 7 to draw a heat map. Finally, the Venn diagrams of the common upregulated and downregulated DEGs in the two data sets is illustrated using the online Venn diagram production website bioinfogp (https://bioinfogp.cnb.csic.es/tools/venny/index.html) [16].

### Gene Ontology (GO) and Kyoto Encyclopedia of Genes and Genomes (KEGG) pathway enrichment analysis

The online database DAVID (DAVID Bioinformatics Resources 6.8) was used to analyze the function and pathway enrichment of the upregulated and downregulated common differentially expressed genes obtained from the intersection of the two chip data sets to further explore the potential pathogenesis of the disease. The upregulated and downregulated differentially expressed genes are imported according to the set differential gene screening criteria from the data after GEO2R analysis and processing into the online enrichment analysis website DAVID (https://david.ncifcrf.gov/tools.jsp) for Gene Ontology (GO) and Kyoto Encyclopedia of Genes and Genomes (KEGG) signal pathway analysis [17, 18]. The GO analysis mainly includes the cellular components (CC), molecular functions (MF), and biological processes (BP) of differential genes.

### Protein-protein interaction (PPI) network analysis

The online analysis website STRING was used to construct a protein-protein interaction network (protein-protein interaction, PPI). The screened upregulated and downregulated common differentially expressed genes were uploaded to the STRING online analysis website (https://string-db.org/); “highest confidence > 0.700” was set in the lowest interaction score, and the protein-protein interaction result data were exported. Furthermore, the protein-protein interaction network (PPI network) of differentially expressed genes was obtained using Cytoscape 3.7.2 software [19], and the cytoHubba plug-in was adopted to screen Hub genes [20]. Meanwhile, three different algorithms (MNC, Degree, and MCC) were used to calculate the top 10 upregulated differentially expressed genes and the top 10 downregulated differentially expressed genes in their respective scores. Besides, the key genes are obtained by the intersection of the gene sets obtained by three different algorithms with the online analysis platform Draw Venn Diagram (http://bioinformatics. psb.ugent.be/webtools/Venn/).

### Peripheral serum collection

From August 2020 to December 2020, peripheral serum samples from 12 steroid-induced ONFH patients (5 males and 7 females; mean age 33.5 ± 9.16) and 8 healthy adults (3 males and 5 females; mean age:32.5 ± 5.9) in the First Affiliated Hospital of Guangzhou University of Chinese Medicine were collected to determine mRNA levels. Two experienced orthopedic surgeons diagnosed the patient with ONFH. To avoid bias, the process of patient selection and matching has been determined prior to the start of the study. The study protocol was reviewed and approved by the Committees of Clinical Ethics of the First Affiliated Hospital of Guangzhou University of Chinese Medicine. Each participant signed the informed consent documents.

### Quantitative real-time PCR analysis

Total RNA from peripheral serum samples was isolated with Trizol LS. The protocol for cDNA synthesis was reverse transcription at 37 °C for 15 min and at 85 °C for 5 s. The qPCR protocol was 95 °C for 30 s, 40 cycles at 95 °C for 5 s, and 60 °C for 30 s. The relative expression level was determined as targeting genes divided by β-Actin (ACTB). Relative miRNA expression was generated with the 2^−△△Cq^ method. Primers used in this study are presented in Table 2. All experiments were performed independently in triplicate.

**Table 2:** The information of the primers’ sequencing

Gene	Forward primers	Reverse primers
TYROBP	TGGTGCTGACAGTGCTCATTGC	AGGCGACTCGGTCTCAGTGATAC
MAPK1	ATGGTGTGCTCTGCTTATGATA	TCTTTCATTTGCTCGATGGTTG
CXCR1	CTGGGAAATGACACAGCAAAAT	GAATCCATAGCAGAACAGCATG
FPR1	CTGTCAGTTATGGGCTTATTGC	GCAATAACTCACGGATTCTGAC
FOXO3	AGCCGAGGAAATGTTCGTC	CCTTATCCTTGAAGTAGGGCAC
FPR2	AACTTCTCCACTCCTCTGAATG	TAATGTGGCCGTGAAAGAAAAG
CXCR2	AAGGTGAATGGCTGGATTTTTG	CCCAGATGCTGAGACATATGAA
ACTB	TGGCACCCAGCACAATGAA	CTAAGTCATAGTCCGCCTAGAAGCA

### Statistical analysis

GraphPad 8.0 was used to perform statistical analysis. The results are presented as mean ± standard deviation (mean ± SD). Data conforming to normal distribution were compared using Student *t* test, while those with non-normally distributed were tested using Mann-Whitney *U* test. *P* < 0.05 indicates statistical significance. All experiments were performed in triplicate.

## Results

### Identification of DEGs

**Fig. 1:**
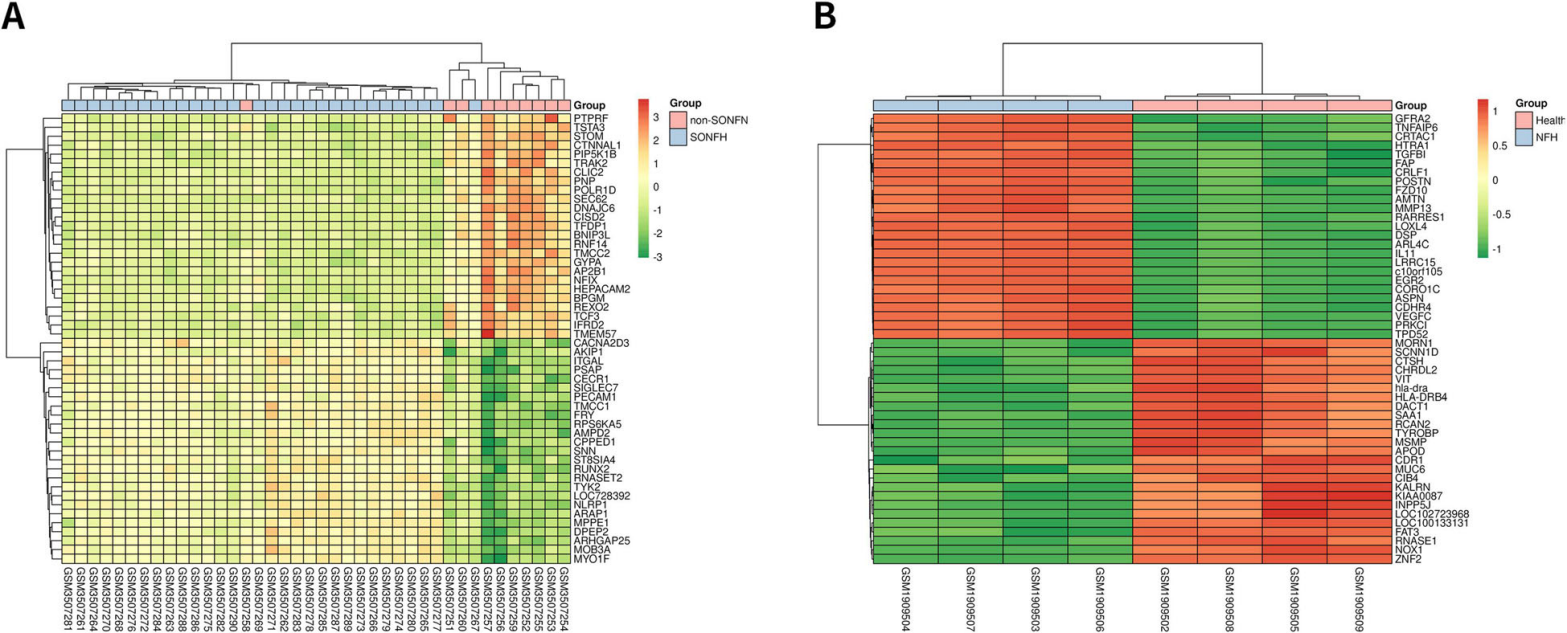
Heat map of top 50 DEGs in ONFH. **a** Heat map of top 50 DEGs in GSE123568. **b** Heat map of top 50 DEGs in GSE74089 (tissue samples are presented as columns; individual genes are presented as rows. Red represents upregulated genes; green indicates downregulated genes in patients with ONFH. The patients in the top rows in pink indicate the control columns; the patients in the top rows in blue indicate columns were patients with NFH. DEGs, differentially expressed genes; NFH, necrosis of the femoral head)

**Fig. 2:**
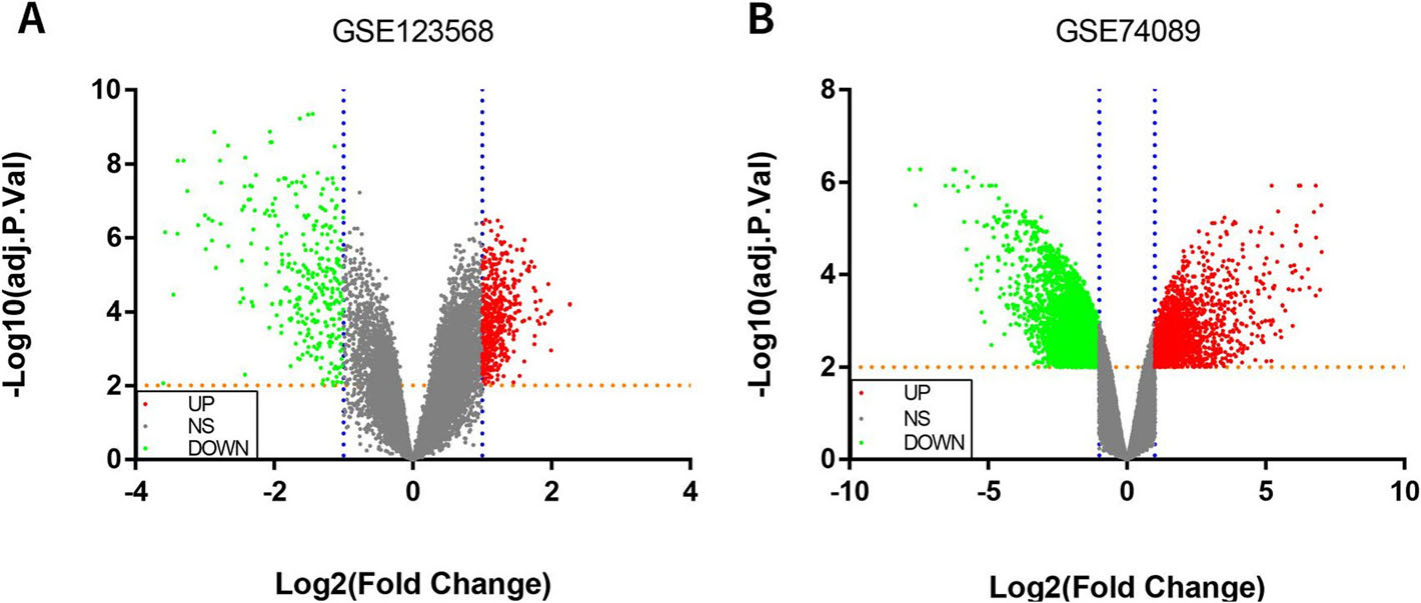
The volcano plot was constructed using fold-change (Log_2_FC) values and adjust *P* values. **a** The volcano plot of a total of 750 DEGs in GSE123568. **b** The volcano plot of total 5211 DEGs in GSE74089 (the vertical blue lines correspond to upregulated and downregulated Log_2_FC; the horizontal Orange line represents an adjust *P* value of 0.01. The red point represents the differentially upregulated genes with statistical significance; the green point suggests the differentially downregulated genes with statistical significance)

**Fig. 3:**
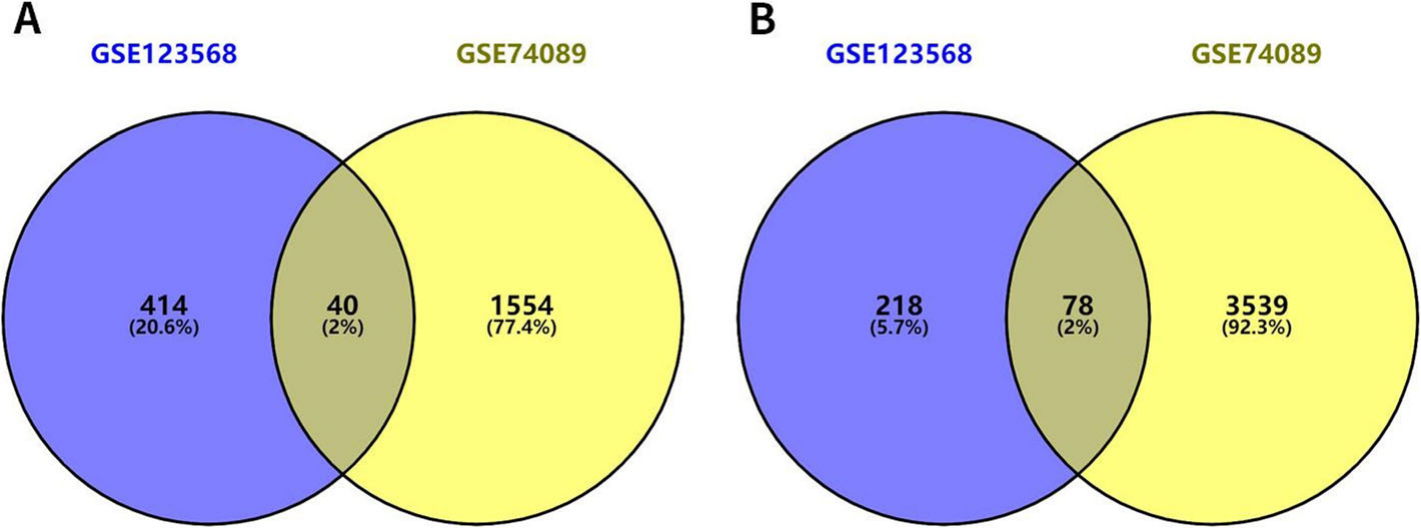
Venn diagrams showing the overlap of DEGs between peripheral serum samples and hip articular cartilage samples. **a** Upregulated DEGs. **b** Downregulated DEGs (Venn diagram was drawn using the online tool (https://bioinfogp.cnb.csic.es/tools/venny/index.html). Numbers represented the number of DEGs. The percentage was represented by the ratio of current DEGs to the total)

This study screened out two chip data sets (GSE123568 and GSE74089). Among them, 30 serum samples of patients with steroid hormone-induced osteonecrosis of the femoral head in the chip data set GSE123568 (including 13 males and 17 females, aged 23.07 ± 3.01 years, ARCO stage I-II patients accounted for 10 patients, ARCO III patients accounted for 10 cases, ARCO IV patients accounted for 10 cases) and 10 normal serum samples (including 7 males and 3 females, aged 25.02 ± 2.87 years). Besides, there are 4 hip cartilage samples from patients with femoral head necrosis (including 3 males and 1 female) and 4 healthy adult hip cartilage specimens (including 3 males and 1 female) in the chip dataset GSE74089. The baseline characteristics of the patients are provided in Table 3. According to the adjust *P* value < 0.01, the expression change range is greater than or equal to twice (|log2 FC|≥1.0) as the criteria for screening differential genes. A total of 750 differentially expressed genes were screened in the GSE123568 data set, including 454 upregulated genes and 296 downregulated genes. A total of 5211 differentially expressed genes were screened in the GSE74089 data set, including 1594 upregulated genes and 3617 downregulated genes. The DEGs in GSE123568 and GSE74089 were selected based on the adjust *P* value to screen the top 25 most significant upregulated and downregulated differentially expressed genes, so as to draw heat maps (Fig. 1). The -log10 conversion is performed for the adjust *P* value in the data processed by GEO2R. Then, according to log2 FC, -log10 (adjust *P* value) was divided into groups of upregulated genome, downregulated genome, and non-statistically different genome. The processed data was imported into GraphPad Prism 7 to draw a volcano map (Fig. 2). Furthermore, a total of 118 intersecting DEGs were obtained between the peripheral blood and cartilage samples, including 40 upregulated genes and 78 downregulated genes (Fig. 3).

**Table 3:** Characteristics of study subjects

GSE12356	Number of subjects	Age (years, mean ± SD)	Sex		ARCO(I–II/III/IV/)
			Male	Female	
**Control**	10	25.02 ± 2.87	7	3	
**Disease**	30	23.07 ± 3.01	13	17	10/10/10
**GSE74089**					
**Control**	4	46.75 ± 4.02	3	1	−
**Disease**	4	45.25 ± 4.02	3	1	−

### Enrichment analysis of KEGG pathway and GO terms

**Fig. 4:**
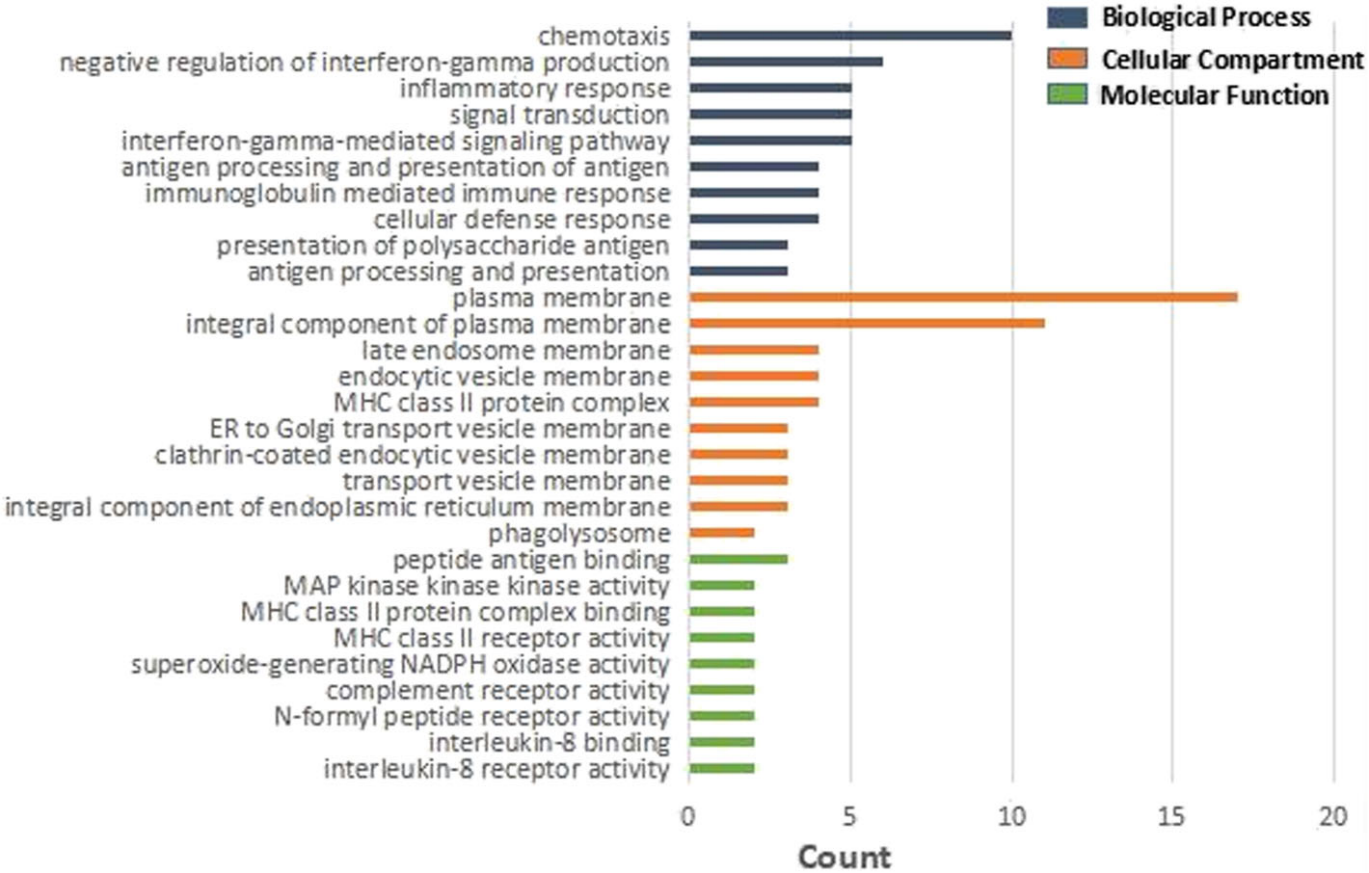
The significant terms identified by GO enrichment analysis for the upregulated DEGs in necrosis of the femoral head samples (*p*<0.05)

**Fig. 5:**
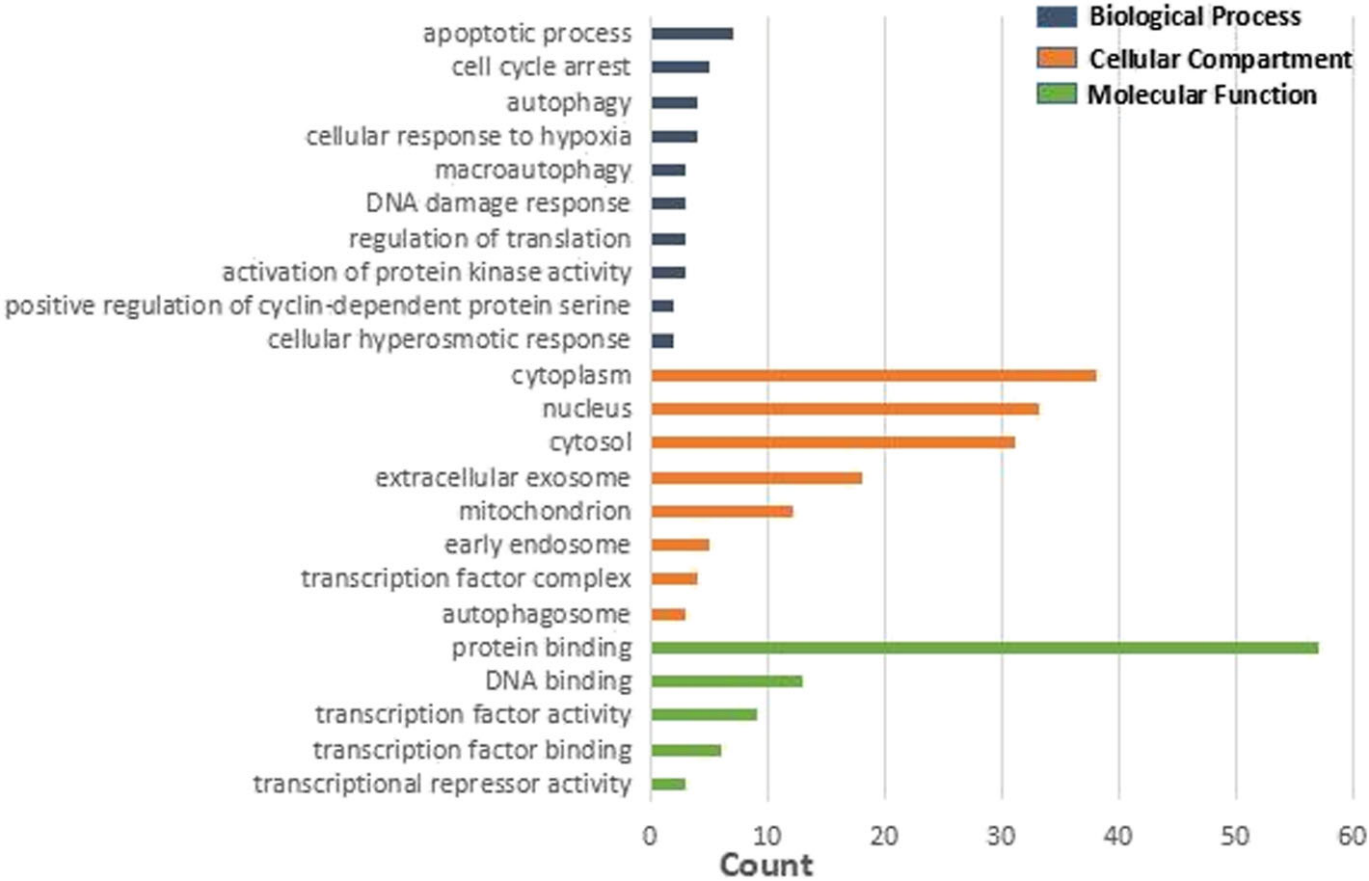
The significant terms identified by GO enrichment analysis for the downregulated DEGs in necrosis of the femoral head samples (*p*<0.05)

**Fig. 6:**
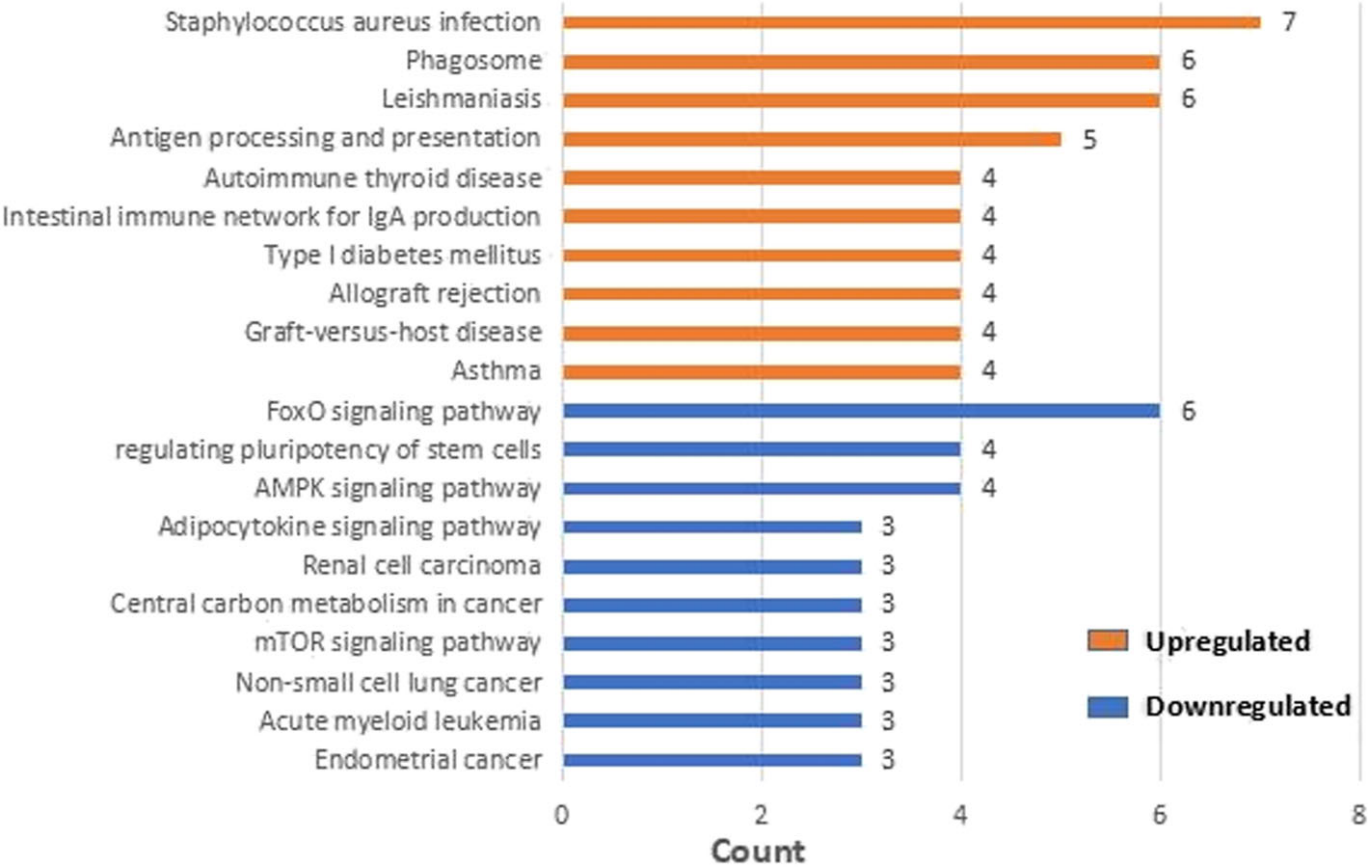
Results of KEGG pathway enrichment analysis of differentially expressed genes

In this study, the DAVID database was used to perform GO and KEGG pathway enrichment analysis on the upregulated and downregulated genes. GO analysis of upregulated DEGs demonstrates that the biological process (BP) is concentrated in inflammation, immune response, and signal transduction. Cellular component (CC) focuses on plasma membrane, endoplasmic reticulum membrane, and lysosome. Molecular functions (MF) are manifested in immune regulation process, IL-8 receptor activity, superoxide-producing NADPH oxidase activity, and MAP kinase activity (Table 4, Fig. 4). Besides, GO analysis of downregulated genes revealed that BP is rich in cell cycle regulation, apoptosis process, translation process regulation, autophagy, and cell response to hypoxia. CC is mainly condensed in cytoplasm, nucleus, mitochondria, autophagosomes, and exosomes. MF is manifested in protein binding, transcription factor binding, and DNA binding. The GO analysis of the top 10 downregulated genes with the smallest adjust *P* value is illustrated in Table 5 and Fig. 5. Enrichment analysis of the upregulated gene KEGG pathway revealed that it focuses on the signaling pathways related to staphylococcus aureus infection, leishmaniasis, antigen processing and presentation, asthma, and graft-versus-host disease. Downregulated genes are concentrated in the FoxO signaling pathway, AMPK signaling pathway, signaling pathway regulating stem cell pluripotency, and mTOR signaling pathway (Table 6, Fig. 6).

**Table 4:** The significant terms identified by GO enrichment analysis for the upregulated DEGs in necrosis of the femoral head samples (*p*<0.05)

Category	Term	Description	Count	***P*** value	Genes
**BP**	GO:0019882	Antigen processing and presentation	5	0.000006999	HLA-DRB5, HLA-DMB, CTSH, HLA-DRB3, HLA-DRB1
	GO:0002504	Antigen processing and presentation of peptide or polysaccharide antigen via MHC class II	4	0.000007113	HLA-DRB5, HLA-DMB, HLA-DRB3, HLA-DRB1
	GO:0006968	Cellular defense response	5	0.000011319	TYROBP, NCF1, NCF2, CXCR2, MNDA
	GO:0002381	Immunoglobulin production involved in immunoglobulin mediated immune response	3	0.000049653	HLA-DRB5, HLA-DRB1, GAPT
	GO:0019886	Antigen processing and presentation of exogenous peptide antigen via MHC class II	5	0.000054005	HLA-DRB5, HLA-DMB, IFI30, HLA-DRB3, HLA-DRB1
	GO:0060333	Interferon-gamma-mediated signaling pathway	4	0.000549531	HLA-DRB5, IFI30, HLA-DRB3, HLA-DRB1
	GO:0007165	Signal transduction	10	0.000916089	CSF3R, TYROBP, CXCR2, FPR1, RARA, ARAP1, STK4, HLA-DRB3, ...
	GO:0006954	Inflammatory response	6	0.001551430	CXCR1, CXCR2, FPR1, CHI3L1, FPR2, AIF1
	GO:0032689	Negative regulation of interferon-gamma production	3	0.001816240	HLA-DRB5, RARA, HLA-DRB1
	GO:0006935	Chemotaxis	4	0.002622088	CXCR1, CXCR2, FPR1, FPR2
**CC**	GO:0042613	MHC class II protein complex	4	0.000013567	HLA-DRB5, HLA-DMB, HLA-DRB3, HLA-DRB1
	GO:0030666	Endocytic vesicle membrane	4	0.000377723	CD163, HLA-DRB5, HLA-DRB3, HLA-DRB1
	GO:0005887	Integral component of plasma membrane	11	0.000608833	CD163, ENTPD1, CSF3R, TYROBP, CXCR2, FPR1, LPAR2, FPR2, ...
	GO:0031902	Late endosome membrane	4	0.001306338	HLA-DRB5, HLA-DMB, HLA-DRB3, HLA-DRB1
	GO:0071556	Integral component of luminal side of endoplasmic reticulum membrane	3	0.001746841	HLA-DRB5, HLA-DRB3, HLA-DRB1
	GO:0030658	Transport vesicle membrane	3	0.002988198	HLA-DRB5, HLA-DRB3, HLA-DRB1
	GO:0030669	Clathrin-coated endocytic vesicle membrane	3	0.003471454	HLA-DRB5, HLA-DRB3, HLA-DRB1
	GO:0012507	ER to Golgi transport vesicle membrane	3	0.005531042	HLA-DRB5, HLA-DRB3, HLA-DRB1
	GO:0005886	Plasma membrane	17	0.007697000	CD163, HLA-DRB5, ENTPD1, CSF3R, FPR1, LPAR2, LILRA1, FPR2, ...
	GO:0032010	Phagolysosome	2	0.008533401	NCF1, NCF2
**MF**	GO:0042605	Peptide antigen binding	3	0.001704631	HLA-DRB5, HLA-DRB3, HLA-DRB1
	GO:0004918	Interleukin-8 receptor activity	2	0.004378952	CXCR1, CXCR2
	GO:0019959	Interleukin-8 binding	2	0.006561426	CXCR1, CXCR2
	GO:0004982	N-formyl peptide receptor activity	2	0.008739245	FPR1, FPR2
	GO:0004875	Complement receptor activity	2	0.013080956	FPR1, FPR2
	GO:0016175	Superoxide-generating NADPH oxidase activity	2	0.023854442	NCF1, NCF2
	GO:0032395	MHC class II receptor activity	2	0.032390762	HLA-DRB3, HLA-DRB1
	GO:0023026	MHC class II protein complex binding	2	0.034513467	HLA-DMB, HLA-DRB1
	GO:0004709	MAP kinase activity	2	0.047154872	MAP3K7CL, MAP3K5

**Table 5:** The significant terms identified by GO enrichment analysis for the downregulated DEGs in necrosis of the femoral head samples (*p*<0.05)

Category	Term	Description	Count	***P***-value	Genes
**BP**	GO:0007050	Cell cycle arrest	5	0.002699248	STK11, CDKN2C, GADD45A, STRADB, PA2G4
	GO:0071456	Cellular response to hypoxia	4	0.007223815	BNIP3L, RGCC, FOXO3, AQP1
	GO:0032147	Activation of protein kinase activity	3	0.014701889	STK11, RGCC, STRADB
	GO:0006914	Autophagy	4	0.017407385	GABARAPL2, STK11, MAP1LC3B, OPTN
	GO:0006417	Regulation of translation	3	0.019332720	AKT2, PA2G4, FOXO3
	GO:0071474	Cellular hyperosmotic response	2	0.024406307	YBX3, AQP1
	GO:0006977	DNA damage response, signal transduction by p53 class mediator resulting in cell cycle arrest	3	0.026857056	TFDP1, RGCC, GADD45A
	GO:0006915	Apoptotic process	7	0.028790620	KANK2, GADD45A, AKT2, SIAH2, PIM1, MAPK1, SUDS3
	GO:0031659	Positive regulation of cyclin-dependent protein serine/threonine kinase activity involved in G1/S tr	2	0.032410549	RGCC, PIM1
	GO:0016236	Macroautophagy	3	0.039024308	GABARAPL2, MAP1LC3B, OPTN
**CC**	GO:0005829	Cytosol	31	0.000004220	SLC2A1, PCTP, HBD, FOXO3, SNX3, STK11, MAP1LC3B, PNP, ...
	GO:0005737	Cytoplasm	38	0.000058040	KANK2, WDR26, ZFAND5, FOXO3, YBX3, TPGS2, TRAK2, AQP1, ...
	GO:0005634	Nucleus	33	0.007200508	DCUN1D1, FOXO3, YBX3, DPCD, TRAK2, AQP1, STK11, PNP, ...
	GO:0005769	Early endosome	5	0.013450748	SNX3, AKT2, SIAH2, MAPK1, TRAK2
	GO:0005739	Mitochondrion	12	0.016407951	KANK2, BNIP3L, ARG2, STK11, MAP1LC3B, TFDP1, BSG, C10ORF10, ...
	GO:0005776	Autophagosome	3	0.027193553	GABARAPL2, MAP1LC3B, OPTN
	GO:0005667	Transcription factor complex	4	0.042391936	HES6, TFDP1, TCF3, PBX1
	GO:0070062	Extracellular exosome	18	0.049903125	ST13, AKR1C1, SLC2A1, CYBRD1, PA2G4, BPGM, PTPRF, AQP1, ...
**MF**	GO:0005515	Protein binding	57	0.000001107	ZFAND5, SLC2A1, CISD2, PCTP, HBD, AQP1, STK11, MAP1LC3B, ...
	GO:0008134	Transcription factor binding	6	0.006463884	HES6, TFDP1, PIM1, MAPK1, TCF3, PBX1
	GO:0001227	Transcriptional repressor activity, RNA polymerase II transcription regulatory region sequence-specific DNA binding	3	0.024911797	HES6, FOXO3, YBX3
	GO:0003677	DNA binding	13	0.041407275	HES6, ZFAND5, PA2G4, FOXO3, KLF3, YBX3, PBX1, TFDP1, ...
	GO:0003700	Transcription factor activity, sequence-specific DNA binding	9	0.044742463	HES6, TFDP1, TCF3, PA2G4, SOX6, FOXO3, YBX3, KLF3, ...

**Table 6:** Results of KEGG pathway enrichment analysis of differentially expressed genes (*p*<0.05)

Category	Term	Description	Count	***P*** value	Genes
**Upregulated genes**	hsa05150	Staphylococcus aureus infection	7	0.000000016	CFD, HLA-DRB5, HLA-DMB, FPR1, FPR2, HLA-DRB3, HLA-DRB1
	hsa05140	Leishmaniasis	6	0.000002958	HLA-DRB5, HLA-DMB, NCF1, NCF2, HLA-DRB3, HLA-DRB1
	hsa04612	Antigen processing and presentation	5	0.000103901	HLA-DRB5, HLA-DMB, IFI30, HLA-DRB3, HLA-DRB1
	hsa04145	Phagosome	6	0.000112986	HLA-DRB5, HLA-DMB, NCF1, NCF2, HLA-DRB3, HLA-DRB1
	hsa05310	Asthma	4	0.000125000	HLA-DRB5, HLA-DMB, HLA-DRB3, HLA-DRB1
	hsa05332	Graft-versus-host disease	4	0.000166885	HLA-DRB5, HLA-DMB, HLA-DRB3, HLA-DRB1
	hsa05330	Allograft rejection	4	0.000235599	HLA-DRB5, HLA-DMB, HLA-DRB3, HLA-DRB1
	hsa04940	Type I diabetes mellitus	4	0.000344316	HLA-DRB5, HLA-DMB, HLA-DRB3, HLA-DRB1
	hsa04672	Intestinal immune network for IgA production	4	0.000481057	HLA-DRB5, HLA-DMB, HLA-DRB3, HLA-DRB1
	hsa05320	Autoimmune thyroid disease	4	0.000648540	HLA-DRB5, HLA-DMB, HLA-DRB3, HLA-DRB1
**Downregulated genes**	hsa04068	FoxO signaling pathway	6	0.000398419	GABARAPL2, STK11, GADD45A, AKT2, MAPK1, FOXO3
	hsa04152	AMPK signaling pathway	4	0.020600369	STK11, AKT2, STRADB, FOXO3
	hsa05213	Endometrial cancer	3	0.025486177	AKT2, MAPK1, FOXO3
	hsa04550	Signaling pathways regulating pluripotency of stem cells	4	0.028841590	PCGF5, AKT2, MAPK1, TCF3
	hsa05221	Acute myeloid leukemia	3	0.029250172	AKT2, PIM1, MAPK1
	hsa05223	Non-small cell lung cancer	3	0.029250172	AKT2, MAPK1, FOXO3
	hsa04150	mTOR signaling pathway	3	0.031210886	STK11, AKT2, MAPK1
	hsa05230	Central carbon metabolism in cancer	3	0.037394653	AKT2, SLC2A1, MAPK1
	hsa05211	Renal cell carcinoma	3	0.039552861	AKT2, SLC2A1, MAPK1
	hsa04920	Adipocytokine signaling pathway	3	0.044008403	STK11, AKT2, SLC2A1

### Construction of PPI network and identification of hub genes

**Fig. 7:**
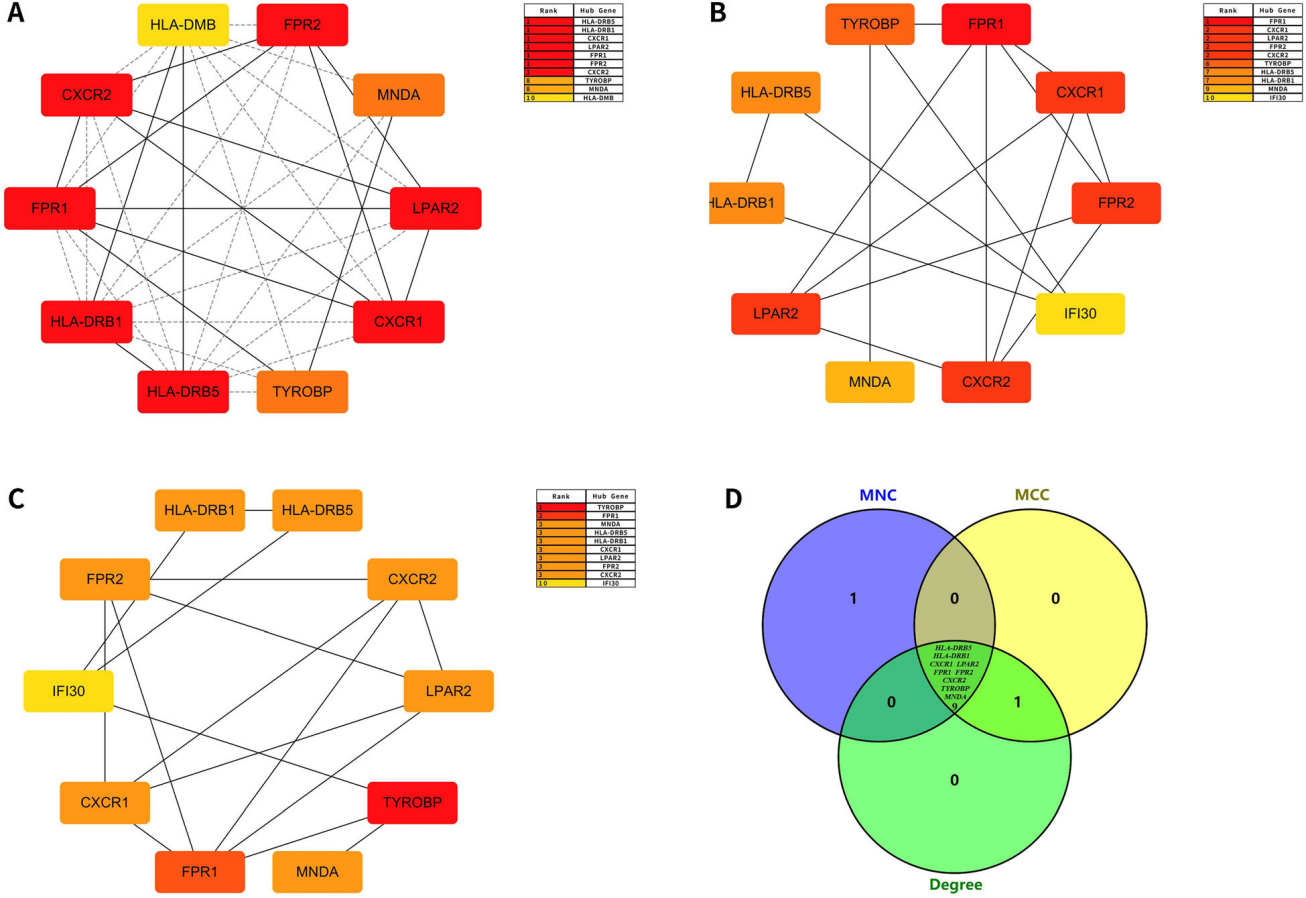
Module identified from the PPI network of the top 10 hub proteins of upregulated DEGs based on MNC centrality, MCC centrality, and degree centrality. **a** The PPI network of the top 10 hub proteins of upregulated DEGs based on MNC centrality; **b** the PPI network of the top 10 hub proteins of upregulated DEGs based on MCC centrality; **c** the PPI network of the top 10 hub proteins of upregulated DEGs based on degree centrality; **d** Venn diagrams of the overlap of upregulated DEGs among MNC centrality, MCC centrality, and degree centrality

**Fig. 8:**
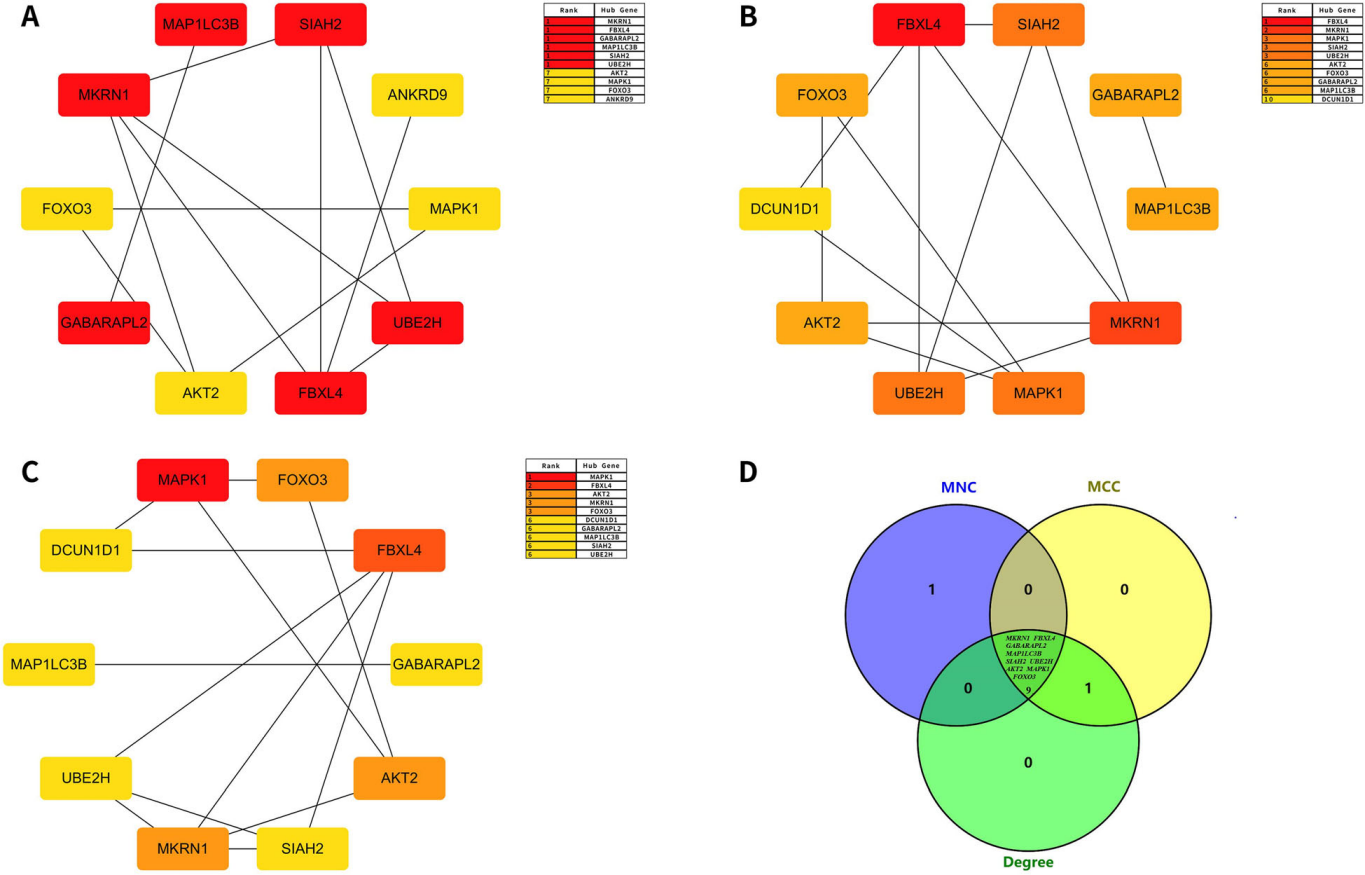
Module identified from the PPI network of the top 10 hub proteins of downregulated DEGs based on MNC centrality, MCC centrality, and degree centrality. **a** The PPI network of the top 10 hub proteins of downregulated DEGs based on MNC centrality; **b** the PPI network of the top 10 hub proteins of downregulated DEGs based on MCC centrality; **c** the PPI network of the top 10 hub proteins of downregulated DEGs based on degree centrality; **d** Venn diagrams of the overlap of downregulated DEGs among MNC centrality, MCC centrality, and degree centrality

Three different algorithms (MNC, MCC, and Degree) in the Cytoscape software’s cytoHubba plug-in were used to calculate top 10 upregulated key genes and top 10 downregulated key genes (Table 7 and Table 8, Fig. 7 and Fig. 8) and obtain the intersection. Hub genes for bone necrosis are as follows: (1) upregulated genes include HLA-DRB5, HLA-DRB1, CXCR1, LPAR2, FPR1, FPR2, CXCR2, TYROBP, and MNDA and (2) downregulated genes include MKRN1, FBXL4, GABARAPL2, MAP1LC3B, SIAH2, UBE2H, AKT2, MAPK1, and FOXO3.

**Table 7:** Evaluation of the top 10 upregulated DEGs of the protein-protein interaction network by MNC centrality, MCC centrality, and degree centrality

Rank	MNC centrality	MCC centrality	Degree centrality
1	HLA-DRB5	FPR1	FPR2
2	HLA-DRB1	CXCR1	CXCL8
3	CXCR1	LPAR2	PF4
4	LPAR2	FPR2	LPAR2
5	FPR1	CXCR2	PSAP
6	FPR2	TYROBP	FPR1
7	CXCR2	HLA-DRB5	CYBB
8	TYROBP	HLA-DRB1	LYN
9	MNDA	MNDA	CBL
10	HLA-DMB	IFI30	CD93

**Table 8:** Evaluation of the top 10 downregulated DEGs of the protein-protein interaction network by MNC centrality, MCC centrality, and degree centrality

Rank	MNC centrality	MCC centrality	Degree centrality
1	MKRN1	FBXL4	MAPK1
2	FBXL4	MKRN1	FBXL4
3	GABARAPL2	MAPK1	AKT2
4	MAP1LC3B	SIAH2	MKRN1
5	SIAH2	UBE2H	FOXO3
6	UBE2H	AKT2	DCUN1D1
7	AKT2	FOXO3	GABARAPL2
8	MAPK1	GABARAPL2	MAP1LC3B
9	FOXO3	MAP1LC3B	SIAH2
10	ANKRD9	DCUN1D1	UBE2H

### Validation of the hub genes in ONFH patients

**Fig. 9:**
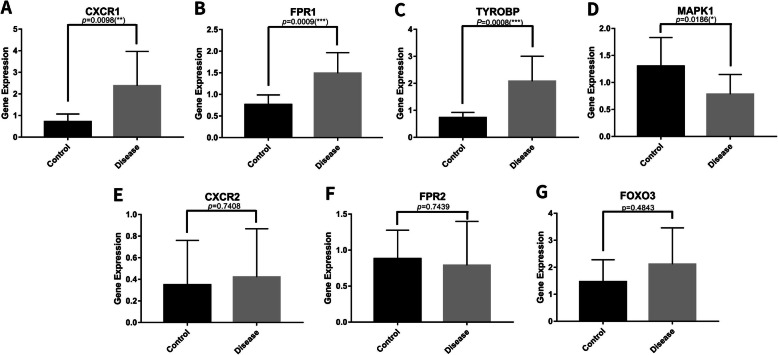
Expression of hub genes in ONFH patients. **a–c** CXCR1, FPR1, and TYROBP were upregulated in peripheral blood of steroid-induced ONFH patients(*P* < 0.05). **d** MAPK1 was downregulated in peripheral blood of steroid-induced ONFH patients (*P* < 0.05); **e**–**g** there was no significant difference in the expression of CXCR2, FPR2, and FOXO3 in peripheral serum samples between the two groups (*P* > 0.05)

Among the identified DEGs, CXCR1, FPR1, MAPK1, FOXO3, FPR2, CXCR2, and TYROBP were selected to verify the integrated result. Peripheral serum samples from 12 steroid-induced ONFH patients and 8 healthy adults were collected to determine mRNA levels. The result revealed that CXCR1, FPR1, and TYROBP were upregulated, and MAPK1 was downregulated in peripheral blood of steroid-induced ONFH patients (*P* < 0.05). There was no significant difference in the expression of FOXO3, FPR2, and CXCR2 in peripheral serum samples between the two groups (*P* > 0.05) (Fig. 9). The expression of CXCR1, FPR1, MAPK1, and TYROBP were consistent with bioinformatics analysis except for FOXO3, FPR2, and CXCR2. Moreover, CXCR1, FPR1, and TYROBP were upregulated, and MAPK1 was downregulated in the peripheral blood of steroid-induced ONFH patients.

## Discussion

Steroid-induced ONFH is a devastating and difficult joint disease and primarily caused by the interruption of blood supply and the dysfunction of the coagulation system, resulting in the death of bone cells, the decrease of the elastic modulus of the femoral head, and the collapse of the femoral head [21]. Various molecular and genetic studies have explored the etiology and pathogenesis [22, 23]. However, the exact pathogenesis remains unclear. In this paper, the genome-wide method was used to study the differential expression of genes in the peripheral blood serum and hip joint cartilage specimens of steroid-induced ONFH patients and healthy controls. Through bioinformatics analysis of GSE123568 and GSE74089, potentially important genes in ONFH were identified. In addition, the role of the identified genes in ONFH and their interaction were analyzed. A total of 118 intersecting DEGs were obtained in peripheral blood and cartilage samples, of which 40 were upregulated genes and 78 were downregulated genes. These DEGs may be important biomarkers related to the pathogenesis and progression of ONFH. Finally, qRT-PCR verified the expression patterns of several genes such as CXCR1, FPR1, MAPK1, FOXO3, FPR2, CXCR2, and TYROBP in vitro. However, due to different inclusion criteria and a small number of patients in the validation set, some results are inconsistent with microarray analysis.

The DEGs screened in this study contribute to the identification of new molecules or new pathways related to steroid-induced ONFH. These new molecules or pathways may be essential targets for disease diagnosis and treatment. The upregulated DEGs were generally involved in inflammation, immune response, and signal transduction. Molecular functions were manifested in immune regulation process, IL-8 receptor activity, superoxide-producing NADPH oxidase activity, and MAP kinase activity. The upregulated DEGs were enriched in pathways engaged in staphylococcus aureus infection, leishmaniasis, antigen processing and presentation, asthma, and graft-versus-host disease. Particularly, the background of this femoral head necrosis can be understood using corticosteroids to treat related acute or chronic graft-versus-host disease (aGVHD, cGVHD) [24]. Socie et al. [25] retrospectively analyzed the risk factors of osteonecrosis after allogeneic hematopoietic stem cell transplantation. The study revealed that the odds ratios of aGVHD and cGVHD were 3.73 and 3.52, respectively. The severity of graft-versus-host disease was associated with a higher frequency of osteonecrosis. Additionally, GO analysis of downregulated genes demonstrated that BP focused on cell cycle regulation, apoptosis process, translation process regulation, autophagy, and cell response to hypoxia. MF was manifested in protein binding, transcription factor binding, and DNA binding. The downregulated genes were concentrated in the FoxO signaling pathway, AMPK signaling pathway, signaling pathway regulating stem cell pluripotency, and mTOR signaling pathway. Moreover, AMPK was an essential energy state sensor responding to energy supply and demand through coordinated metabolic pathways [26]. Studies have indicated that AMPK can promote cell survival under various stress conditions [27]. The forced activation of AMPK is a good strategy to protect osteoblasts from Dex [28, 29]. The mTOR pathway may be a vital mediator of bone homeostasis [30]. Furthermore, the impaired differentiation of the osteogenic/adipogenic lineage of bone marrow mesenchymal stem cells (BMSCs) was caused by the activation of the mTOR signaling pathway [31]. Meanwhile, blocking the mTOR pathway can prevent the development of phenotypes related to aging and enhance the proliferation ability of BMSCs [32]. Therefore, regulating the mTOR signaling pathway may be one of the crucial methods to treat steroid-induced ONFH.

The construction of the PPI network of DEGs was used to study the relationship between GO enrichment and important proteins identified by pathway analysis. The upregulated DEGs included TYROBP, CXCR1, FPR1, LPAR2, FPR2, HLA-DRB5, HLA-DRB1, CXCR2, and MNDA. TYROBP is a protein, which is involved in osteoclast differentiation and function, such as the generation of actin cytoskeleton, which is very important for bone resorption [33]. In this study, both biosynthesis analysis and specimen verification found that the expression of TYROBP was upregulated, indicating that TYROBP may play an important role in regulating osteoclast differentiation in steroid-induced ONFH. Specifically, CXCR1 (IL 8 receptor), a chemokine, is recognized to be abundant in the synovial fluid of bone marrow mesenchymal stem cells and has been verified to be a potent inducer of bone marrow mesenchymal stem cell migration [33]. Chemokines can induce the migration response of bone marrow mesenchymal stem cells to cartilage defect areas [34]. Hence, CXCR1 may play an important role in the repair of steroid-induced ONFH cartilage. Besides, FPR is a chemotactic receptor, and its expression is often identified in phagocytes (monocytes and neutrophils, etc.). FPR may play a role in promoting stem cell reproliferation because MSCs functionally express FPR and are positive for n-formyl peptides [35]. Matsushita et al. [36] found an important role of fMLP or FRP1 in MSC osteogenic and adipogenic differentiation. Moreover, the important role of fMLP is to promote the osteoblast differentiation of MSCs, and the knockdown of FPR1 inhibits this osteogenic differentiation. Downregulated DEGs identified in the present study included MAPK1, AKT2, MKRN1, FBXL4, GABARAPL2, MAP1LC3B, SIAH2, UBE2H, and FOXO3. MAPK was a type of serine/threonine protein kinase in cells that can regulate cell growth and differentiation. Experiments have demonstrated that activating the MAPK signaling pathway can promote the mineralization of osteoblasts and the expression of ALP and BMP-2, contributing to bone formation. ERK, a downstream factor of MAPK, can inhibit osteoclast formation when it is inactivated [35]. We discovered that MAPK1 was downregulated in steroid-induced ONFH blood samples compared with normal controls, providing another pathogenic role in bone disease. Moreover, AKT2 is a pro-survival protein in the AKT family and is activated through the PI3K pathway. Zhang et al. [37] revealed that AKT2 plays a crucial role in the development of zebrafish bone. Furthermore, inhibiting the PI3k/Akt signaling pathway may be one of the vital mechanisms of glucocorticoid-induced osteoblast apoptosis [38]. In the present study, the decreased expression of AKT2 was observed in steroid-induced ONFH, suggesting a vital role in bone formation and metabolism.

There are limitations to our study. First, the sample size in the qRT-PCR data set was small, and larger numbers of blood samples of ONFH patients are needed for further research. Second, the genes investigated and their pathways were not confirmed through in vitro studies or other functional studies. Therefore, the potential role of DEGs and PPIs identified in ONFH patients requires to be further investigated in vivo and in vitro. For example, the effects of downregulated or upregulated DEGs in cellular or in vivo models could be determined.

In conclusion, the present study would provide novel insight into the genes and associated pathways involved in steroid-induced ONFH. CXCR1, FPR1, TYROBP, and MAPK1 could serve as potential drug targets and biomarkers for the diagnosis and prognosis of steroid-induced ONFH.

## Data Availability

All the data will be available upon motivated request to the corresponding author of the present paper.

## Abbreviations

ONFH: Osteonecrosis of the femoral head
KEGG: Kyoto Encyclopedia of Genome
GO: Gene Ontology
PPI: Protein-protein interaction
NCBI: National Center for Biotechnology Information

## References

[1] Zhang QY, Li ZR, Gao FQ, Sun W (2018). Pericollapse stage of osteonecrosis of the femoral head: a last chance for joint preservation. Chin Med J (Engl).

[2] Song Y, Du Z, Ren M (2017). Association of gene variants of transcription factors PPARγ, RUNX2, Osterix genes and COL2A1, IGFBP3 genes with the development of osteonecrosis of the femoral head in Chinese population. Bone.

[3] Seamon J, Keller T, Saleh J, Cui Q (2012). The pathogenesis of nontraumatic osteonecrosis. Arthritis.

[4] Hauzeur JP, Malaise M, de Maertelaer V (2016). A prospective cohort study of the clinical presentation of non-traumatic osteonecrosis of the femoral head: spine and knee symptoms as clinical presentation of hip osteonecrosis. Int Orthop.

[5] Moya-Angeler J, Gianakos AL, Villa JC, Ni A, Lane JM (2015). Current concepts on osteonecrosis of the femoral head. World J Orthop.

[6] Johnson AJ, Mont MA, Tsao AK, Jones LC (2014). Treatment of femoral head osteonecrosis in the United States: 16-year analysis of the Nationwide Inpatient Sample. Clin Orthop Relat Res.

[7] Mont MA, Cherian JJ, Sierra RJ, Jones LC, Lieberman JR (2015). Nontraumatic osteonecrosis of the femoral head: where do we stand today? A ten-year update. J Bone Joint Surg Am.

[8] Du J, Liu W, Jin T (2016). A single-nucleotide polymorphism in MMP9 is associated with decreased risk of steroid-induced osteonecrosis of the femoral head. Oncotarget.

[9] Gu C, Xu Y, Zhang S, Guan H, Song S, Wang X, Wang Y, Li Y, Zhao G (2016). miR-27a attenuates adipogenesis and promotes osteogenesis in steroid-induced rat BMSCs by targeting PPARγ and GREM1. Sci Rep.

[10] Hao C, Yang S, Xu W, Shen JK, Ye S, Liu X, Dong Z, Xiao B, Feng Y (2016). MiR-708 promotes steroid-induced osteonecrosis of femoral head, suppresses osteogenic differentiation by targeting SMAD3. Sci Rep.

[11] Franco D, Bonet F, Hernandez-Torres F, Lozano-Velasco E, Esteban FJ, Aranega AE (2016). Analysis of microRNA microarrays in cardiogenesis. Methods Mol Biol.

[12] Kang S, Song J (2017). Robust gene selection methods using weighting schemes for microarray data analysis. BMC Bioinformatics.

[13] Magnussen RA, Guilak F, Vail TP (2005). Articular cartilage degeneration in post-collapse osteonecrosis of the femoral head. Radiographic staging, macroscopic grading, and histologic changes. J Bone Joint Surg Am.

[14] Barrett T, Wilhite SE, Ledoux P, Evangelista C, Kim IF, Tomashevsky M, Marshall KA, Phillippy KH, Sherman PM, Holko M, Yefanov A, Lee H, Zhang N, Robertson CL, Serova N, Davis S, Soboleva A (2013). NCBI GEO: archive for functional genomics data sets--update. Nucleic Acids Res.

[15] Metsalu T, Vilo J (2015). ClustVis: a web tool for visualizing clustering of multivariate data using principal component analysis and heatmap. Nucleic Acids Res.

[16] Oliveros JC. Venny. An interactive tool for comparing lists with Venn's diagrams. 2015. https://bioinfogp.cnb.csic.es/tools/venny/index.html.

[17] Huang DW, Sherman BT, Lempicki RA (2009). Bioinformatics enrichment tools: paths toward the comprehensive functional analysis of large gene lists. Nucleic Acids Res.

[18] Huang DW, Sherman BT, Lempicki RA (2009). Systematic and integrative analysis of large gene lists using DAVID bioinformatics resources. Nat Protoc.

[19] Shannon P, Markiel A, Ozier O, Baliga NS, Wang JT, Ramage D, Amin N, Schwikowski B, Ideker T (2003). Cytoscape: a software environment for integrated models of biomolecular interaction networks. Genome Res.

[20] Chin CH, Chen SH, Wu HH (2014). cytoHubba: identifying hub objects and sub-networks from complex interactome. BMC Syst Biol.

[21] Cohen-Rosenblum A, Cui Q (2019). Osteonecrosis of the femoral head. Orthop Clin North Am.

[22] Wei B, Wei W (2015). Identification of aberrantly expressed of serum microRNAs in patients with hormone-induced non-traumatic osteonecrosis of the femoral head. Biomed Pharmacother.

[23] Song Y, Du ZW, Yang QW (2018). Erratum: Association of genes variants in RANKL/RANK/OPG signaling pathway with the development of osteonecrosis of the femoral head in Chinese population: Erratum. Int J Med Sci.

[24] Jones KB, Seshadri T, Krantz R, Keating A, Ferguson PC (2008). Cell-based therapies for osteonecrosis of the femoral head. Biol Blood Marrow Transplant.

[25] Socié G, Cahn JY, Carmelo J, Vernant JP, Jouet JP, Ifrah N, Milpied N, Michallet M, Lioure B, Pico JL, Witz F, Molina L, Fischer A, Bardou VJ, Gluckman E, Reiffers J (1997). Avascular necrosis of bone after allogeneic bone marrow transplantation: analysis of risk factors for 4388 patients by the Société Française de Greffe de Moëlle (SFGM). Br J Haematol.

[26] Carling D, Thornton C, Woods A, Sanders MJ (2012). AMP-activated protein kinase: new regulation, new roles?. Biochem J.

[27] Wang S, Song P, Zou MH (2012). AMP-activated protein kinase, stress responses and cardiovascular diseases. Clin Sci (Lond).

[28] Guo S, Chen C, Ji F, Mao L, Xie Y (2017). PP2A catalytic subunit silence by microRNA-429 activates AMPK and protects osteoblastic cells from dexamethasone. Biochem Biophys Res Commun.

[29] Liu W, Mao L, Ji F, Chen F, Hao Y, Liu G (2017). Targeted activation of AMPK by GSK621 ameliorates H2O2-induced damages in osteoblasts. Oncotarget.

[30] Chen C, Akiyama K, Wang D, Xu X, Li B, Moshaverinia A, Brombacher F, Sun L, Shi S (2015). mTOR inhibition rescues osteopenia in mice with systemic sclerosis. J Exp Med.

[31] Liu Y, Kou X, Chen C, Yu W, Su Y, Kim Y, Shi S, Liu Y (2016). Chronic high dose alcohol induces osteopenia via activation of mTOR signaling in bone marrow mesenchymal stem cells. Stem Cells.

[32] Gharibi B, Farzadi S, Ghuman M, Hughes FJ (2014). Inhibition of Akt/mTOR attenuates age-related changes in mesenchymal stem cells. Stem Cells.

[33] Park MS, Kim YH, Jung Y, Kim SH, Park JC, Yoon DS, Kim SH, Lee JW (2015). In situ recruitment of human bone marrow-derived mesenchymal stem cells using chemokines for articular cartilage regeneration. Cell Transplant.

[34] Koelling S, Kruegel J, Irmer M, Path JR, Sadowski B, Miro X, Miosge N (2009). Migratory chondrogenic progenitor cells from repair tissue during the later stages of human osteoarthritis. Cell Stem Cell.

[35] Matsushita T, Chan YY, Kawanami A, Balmes G, Landreth GE, Murakami S (2009). Extracellular signal-regulated kinase 1 (ERK1) and ERK2 play essential roles in osteoblast differentiation and in supporting osteoclastogenesis. Mol Cell Biol.

[36] Shin MK, Jang YH, Yoo HJ, Kang DW, Park MH, Kim MK, Song JH, Kim SD, Min G, You HK, Choi KY, Bae YS, Min DS (2011). N-formyl-methionyl-leucyl-phenylalanine (fMLP) promotes osteoblast differentiation via the N-formyl peptide receptor 1-mediated signaling pathway in human mesenchymal stem cells from bone marrow. J Biol Chem.

[37] Zhang D, Wang J, Zhou C, Xiao W (2017). Zebrafish akt2 is essential for survival, growth, bone development, and glucose homeostasis. Mech Dev.

[38] Piovan E, Yu J, Tosello V, Herranz D, Ambesi-Impiombato A, da Silva AC, Sanchez-Martin M, Perez-Garcia A, Rigo I, Castillo M, Indraccolo S, Cross JR, de Stanchina E, Paietta E, Racevskis J, Rowe JM, Tallman MS, Basso G, Meijerink JP, Cordon-Cardo C, Califano A, Ferrando AA (2013). Direct reversal of glucocorticoid resistance by AKT inhibition in acute lymphoblastic leukemia. Cancer Cell.

